# Archaeal diversity in the microbiomes of four wild bird species

**DOI:** 10.1128/spectrum.02870-24

**Published:** 2025-03-25

**Authors:** Trevor Hunter, Lauren Chance, Chris S. Elphick, Sarah M. Hird

**Affiliations:** 1 Molecular and Cell Biology Department, University of Connecticut 124501 , Storrs, Connecticut, USA; 2 Ecology and Evolutionary Biology Department, University of Connecticut 164548 , Storrs, Connecticut, USA; 3 Institute for Systems Genomics, University of Connecticut 703792, Storrs, Connecticut, USA; Agriculture and Agri-Food Canada, Lacombe, Canada

**Keywords:** Archaea, birds, microbiome, wildlife

## Abstract

Archaea are generally low-abundance members of the vertebrate microbiota that require specific PCR primers to be detected in metabarcoding studies, and the robust intraspecific sample size is necessary for well-supported conclusions about archaeal diversity. Using 16S rRNA gene amplicons generated using both Archaea-Specific and Universal primers, we investigated prokaryotic diversity in 110 fecal samples from four wild bird species from four different orders: Anna’s Hummingbird (*Calypte anna*), Saltmarsh Sparrow (*Ammospiza caudacuta*), Ruddy Turnstone (*Arenaria interpres*), and Canada Goose (*Branta canadensis*). Our aim was to test the hypotheses that Archaea-Specific primers would offer higher resolution of archaeal diversity and that the four ecologically distinct host species would have distinct archaeal communities. Archaea-Specific primers resulted in increases in archaeal richness and detection of Archaea in all four birds compared to the Universal primers. The ammonia-oxidizing archaeal order *Nitrososphaerales* was detected in all four host species, and methanogenic orders were enriched in samples from Canada Geese. In Bacteria-Archaea co-occurrence networks, Archaea-Specific primers found many more significant interactions than the Universal primers alone. Methanogenic archaeal orders dominated the microbiota in Canada Geese and were found to a lesser extent in the other host species, suggesting an important functional role of methanogens in Canada Geese. Overall, this study advances our knowledge of the archaeal component of the microbiome in wild birds and provides insight into the potential functional roles Archaea play in studies of avian gastrointestinal microbiota.

**IMPORTANCE:**

Archaea may be persistent members of host-associated microbiomes across diverse host taxa; their detection has been limited due to their low abundance and the inadequacy of Universal primers. Large-scale studies of Archaea in vertebrate microbiomes have historically had low intraspecific sample sizes for bird species and had conflicting results. This study demonstrates the improved capability of the Archaea-Specific primers to detect archaeal diversity in diverse avian host species compared to the widely used Universal primers. We also identified both shared and species-specific archaeal taxa across four ecologically distinct avian host species from four different orders with implications for functional importance. Future studies interested in comprehensively cataloging prokaryotic diversity in avian microbiomes using amplicon-based sequencing methods should include Archaea-Specific primers to adequately probe archaeal diversity.

## INTRODUCTION

The microbiome is a community of microorganisms living in a specific environment. When this environment is a multicellular organism, the microbiome is host-associated. Microbiomes have many impacts on their host organisms, such as aiding in host development (1), digestion (2), and immune function (3). In addition to interacting with the host, microorganisms in the microbiome interact with each other. The interactions between microbial organisms and the overall community dynamics of the microbiome are extensively studied in the context of microbial ecology and benefits to host organisms (e.g., (4–7). Most microbiome research focuses on the bacterial members of the community and neglects the archaeal, viral, and microscopic eukaryotic members (8). This is largely due to methodological limitations of the amplicon sequencing that is commonly used to study the taxonomic composition of the microbiome (9–11). To encourage the standardization of 16S rRNA gene amplicon-based research, the Earth Microbiome Project generated a “Universal 16S rRNA gene V4 primer set” to characterize diversity in the variable region 4 of the prokaryotic 16S rRNA gene around the world (12, 13). These primers have been widely adopted by researchers of microbiome studies and can detect both Bacteria and Archaea. However, these Universal primers do not detect the entirety of archaeal diversity in vertebrate gut microbiomes, and reliable amplification of Archaea suffers due to the unequal abundances of Bacteria and Archaea (9–11).

Archaea and Bacteria are both unicellular organisms, but they represent distinct domains of life with different DNA replication machinery and different cell envelope structures (14). Archaea were initially thought to be entirely comprised of extremophiles since they were discovered in environments with extreme conditions, such as high-temperature hot springs and high-salinity salt lakes (15). As more Archaea were found in less extreme environments, it was clear that Archaea are ubiquitously distributed, much like Bacteria and Eukaryotes (16). This distribution includes host-associated microbiomes, where Archaea can be significant members of the microbiota, such as the gastrointestinal tracts of mammalian ruminants like cattle (*Bos taurus*) and in wood-digesting insects, like termites (Isoptera) (17, 18). Research into the function of Archaea in host-microbe symbioses revealed unique archaeal metabolic capabilities such as using CO_2_ and H_2_ produced by fermentative bacteria for methanogenic metabolism (17, 18). This, in turn, benefited host animals by reducing the partial gas pressure of H_2_, preventing inhibition of bacterial fermentation for the fibrous plant matter that the hosts feed on (19). Research into the diversity of Archaea in animal hosts is not as widespread as it is for host-associated Bacteria, and due to the distinct cell biology of these two domains, information about the impact of host-associated microbes on the host is incomplete (10, 11).

Birds are globally important ecologically (20), economically (21), and in relation to human public health, especially with regard to zoonotic diseases like Influenza A (22). The bacterial component of the avian microbiome correlates with age (23), diet (24), geography (25), elevation (26), host phylogeny (27), and captivity (28). However, most studies of avian microbiota either exclude Archaea or Archaea represent so little of the overall sequenced microbiota that their importance may be overlooked. Therefore, relatively little is known about Archaea in the avian microbiome. Large-scale studies of Archaea in vertebrate microbiomes have historically had low intraspecific sample sizes for bird species and have produced conflicting results (10, 11). Youngblut et al. (10) found Archaea from the genus *Methanothermobacter* in 15 out of 21 bird species and hypothesized that this association may be due to the relatively high body temperatures in birds which might facilitate the presence of the thermophilic *Methanothermobacter*, while Thomas et al. (11) found low concentrations of Archaea 16S rRNA gene copies in 42 out of 46 species of birds in their qPCR experiments and proposed that perhaps some birds lack a functional microbiome. Archaea are essential members of some avian microbiota that are likely to contribute to the bird’s ecology. For example, Hoatzins (*Ophisthocomus hoazin*), a foregut fermenting folivore, have a large crop full of digesta and microbes. This microbial community, and the individual archaeal taxa within it, are similar to the cattle (*Bos taurus*) rumen microbiota suggesting that an herbivorous diet requiring bacterial fermentation could be a strong driver of archaeal members of the microbiota (29)￼. Given that Archaea are important components of the microbiota in some avian taxa, it is necessary to broadly survey avian archaeal diversity to enable future research investigating the functional significance of Archaea in avian-associated microbiota.

The methodological aim of this study was to compare archaeal taxonomic diversity in the fecal microbiome of four bird species using two different primer sets targeting the 16S rRNA gene. The Universal primer set, designed by the Earth Microbiome Project, amplifies both Archaea and Bacteria (12, 13). The Archaea-Specific primer set specifically amplifies Archaea (9, 30). The biological aim of this study was to compare the composition and diversity of archaeal taxa across four bird species that have differing natural histories. In addition to investigating the composition and diversity of archaeal taxa, we explore the relationships between archaeal and bacterial taxa in the four bird species. We used co-occurrence networks to look for significant correlations between the presence of archaeal and bacterial taxa. For the methodological aim, we expected that the Archaea-Specific primers would uncover greater archaeal diversity than the Universal primers (9–11). For the biological aim, we hypothesized that each bird species would have both unique and overlapping archaeal taxa in their microbiomes.

This study has a statistically robust (*n* > 20) intraspecific sample size for four species, each representing a different order of birds. These species have varied life-history traits including size, diet, and habitat. Anna’s Hummingbird (*Calypte anna*) is in the order Apodiformes*,* and they inhabit the Pacific coast of North America. They have one of the highest mass-specific metabolic rates of all vertebrates that powers their incredible flight abilities and requires a mostly nectivorous diet that is supplemented with arthropods for protein (31, 32). Canada Goose (*Branta canadensis*) is a member of the order Anseriformes*,* and they are found throughout Canada and the United States. They are one of the few folivorous avian species and exist in both resident and migratory populations (33, 34). Ruddy Turnstone (*Arenaria interpres*) is a shorebird of the order Charadriiformes, and they migrate from their non-breeding grounds to their breeding grounds in the Arctic. During migration, birds rest at stopover sites, such as Delaware Bay, where they gorge themselves on a diet of Horseshoe Crab (*Limulus polyphemus*) eggs (35). Saltmarsh Sparrow (*Ammospiza caudacuta*) is in the order Passeriformes*,* and they inhabit tidal marshes along the Atlantic coast of North America. Populations are declining rapidly due to high nest failure caused by increased flooding rates associated with sea-level rise, and extinction is expected as soon as 2050 (36, 37). These birds have a generalist diet and feed on insects, marine invertebrates, and seeds (38). Based on the natural history and lifestyles of these species, we expected that Canada Geese would have relatively higher abundances of methanogenic Archaea compared to the other three host species due to their primarily folivorous diet. This hypothesis is based on previous results that showed an enrichment of genes and transcripts associated with bacterial carbohydrate digestion in Canada Geese (39). We expected results supporting the potential for degradation of plant carbohydrates by Bacteria and Archaea similar to those found in the Hoatzin (29). In addition, we expect to find Archaea from the ammonia-oxidizing phylum *Nitrososphaera* (formerly *Thaumarchaeota*), as it was found in birds in previous studies (10, 11).

## MATERIALS AND METHODS

### Sample selection and processing

Fecal samples from four bird species were chosen for this study to maximize available diversity in taxonomy and natural history and included Anna’s Hummingbird (*n* = 24), Canada Goose (*n* = 40), Ruddy Turnstone (*n* = 24), and Saltmarsh Sparrow (*n* = 22). Fecal samples from each species were collected, processed, and sequenced separately as they all came from previous studies; however, protocols are largely uniform, as they all come from the same lab group. Slight differences exist between methodologies of the previous studies, and this potentially introduced batch effects that cannot be disentangled from biologically relevant differences in the microbiomes of these four bird species. For three of the focal species, the bacterial component of the microbiome was previously characterized with the Universal primers (Anna’s Hummingbird [26]; Canada Goose [40]; Ruddy Turnstone [41]). In all cases, the same or similar methods were used to process the fecal samples and generate the sequencing libraries.

Characterization of the Saltmarsh Sparrow microbiome using the Universal primers was initiated in this study, with DNA extraction, sample processing, and amplicon sequencing conducted to match the methods in the previous studies. Briefly, DNA from Saltmarsh Sparrow samples was extracted using a modified protocol for the ZymoBiomics DNA miniprep Kit (Zymo Research, Irvine, CA, USA). The provided Lysis buffer was not used and was replaced with an additional ZymoBiomics DNA/RNA shield (Zymo Research, Irvine, CA, USA). The bead beating step was increased to 30 minutes, and a double final elution using 75 μl of DNase/RNase-free water at 60°C was performed. Extracted DNA was stored at −80°C for long-term storage before use in PCR.

### PCR amplification

Only the Saltmarsh Sparrow samples were amplified and sequenced with Universal primers (12, 13) for this study specifically: DNA elutions were amplified using primers specific to the V4 variable region of the 16S rRNA gene and sequenced at the University of Connecticut Microbial Analysis, Resources, and Services (MARS) core lab using standard protocols. In brief, indexed 515F and 806R primers and GoTaq (Promega, Madison, WI, USA) with the addition of 10 mg bovine serum albumin (BSA) were used for PCR amplification. Then, PCR was incubated at 95°C for 2 minutes, the 30 cycles of 30 s at 95.0°C, 60 s at 50.0°C, and 60 s at 72.0°C, followed by a final extension at 72.0°C for 10 minutes. PCR products were quantified and visualized using the QIAxcel DNA Fast Analysis (Qiagen, Hilden, Germany).

All samples were amplified and sequenced with the Archaea-Specific primers (30); DNA elutions were amplified using GoTaq Green Mastermix (Promega, Madison, WI, USA) and primers with index sequences for two-step Illumina library preparation (Illumina, San Diego, CA, USA). Primer sequences were as follows with the index in *italics* and the target sequence in **bold**: Indexed516F (*ACACTCTTTCCCTACACGACGCTCTTCCGATCT***TGYCAGCCGCCGCGGTAAHACCVGC**); Indexed915R (*GTGACTGGAGTTCAGACGTGTGCTCTTCCGATCT***GTGCTCCCCCGCCAATTCCT**). The PCR included an initial incubation at 95°C for 2 minutes, then 30 cycles of 95°C for 60 s, 64°C for 60 s, and 72°C for 60 s and a final extension at 72°C for 5 minutes. Amplification was confirmed using gel electrophoresis.

### Sequencing

The Saltmarsh Sparrow samples prepared with the Universal primers and all samples prepared with the Archaea-Specific primers were sequenced on the Illumina MiSeq (Illumina, San Diego, CA, USA) platform at the University of Connecticut MARS core lab following library preparation. The samples were divided into two sequencing runs, with the first containing all the Canada Goose samples and the second containing all the other samples. Canada Goose samples were run separately as a preliminary proof of concept experiment. Sequencing runs also included PCR and extraction negative controls (*N* = 23. Canada Goose: 4 Extraction, 1 PCR; Anna’s Hummingbird: 1 Extraction, 3 PCR; Saltmarsh Sparrow: 8 Extraction, 2 PCR; Ruddy Turnstone: 2 Extraction, 2 PCR). Negative extraction controls were made during DNA extraction using only storage buffer instead of fecal matter and went through all subsequent steps of the protocol. Negative PCR controls were made during PCR and used only nuclease-free water instead of extracted sample DNA and then went through all subsequent steps of the protocol. The previously and newly generated sequence data are all publicly available (Table 1).

**TABLE 1:** Publicly available sequences

Bird species	Number of samples	Universal citation	Universal primer raw reads	Archaea-Specific primers raw reads
Anna’s Hummingbird	24	(26)	SRA Accession PRJNA659540	SRA Accession PRJNA1219551
Canada Goose	40	(40)	SRA Accession PRJNA906628	SRA Accession PRJNA1219117
Ruddy Turnstone	24	(41)	https://doi.org/10.6084/m9.figshare.11337716.v1	SRA Accession PRJNA1224787
Saltmarsh Sparrow	22	N/A	SRA Accession PRJNA1218777	SRA Accession PRJNA1224780

### Sequence processing

Using the raw data from the previous studies as well as the data generated for this study, sequence libraries for both the Universal and Archaea-Specific amplicons from all four bird species were processed into amplicon sequence variants (ASVs) using DADA2 (v 1.26.0) in R version 4.2.2 (42, 43). Processed sequences were aligned to the SILVA rRNA reference database version 138.1 (44) and assigned taxonomy using the RDP Naive Bayesian Classifier algorithm (45). For the Universal Primers data set, only ASVs corresponding to Archaea and Bacteria were kept; sequences identified as chloroplast or mitochondria represented 15.3% of total reads and were removed. The sample metadata, ASVs, and assigned taxonomy were combined into a phyloseq (v 1.42.0) object (46) for analysis. Potential contaminant sequences were removed using the prevalence method from the decontam package (v 1.18.0) (47) and utilizing the DNA extraction and PCR-negative controls.

### Data analyses

R (version 4.2.2) was used for all analyses (43). All visualizations were made using ggplot2 (v 3.5.1) (48) unless otherwise stated. The relative abundance of ASVs for each sample was calculated for Archaea for both primer sets and for Bacteria for the Universal primers using phyloseq (v 1.42.0) (46) and the microbiome package (v 1.20.0) (49). Relative abundances were visualized as stacked bar graphs. Differential abundance of archaeal ASVs was calculated pairwise between each host species and between the Canada Goose samples and the other three species combined using ANCOM-BC2 with default settings using the ANCOMBC package (v 2.0.3) (50, 51). The comparison between the Canada Goose and the other three species allowed a test with robust sample size as the Canada Goose had 39 individuals and the other three species combined had 36 individuals (Anna’s Hummingbird *n* = 14, Ruddy Turnstone *n* = 19, Saltmarsh Sparrow *n* = 3). Only samples containing reads assigned to Archaea using the Archaea-Specific 16S rRNA PCR primers were used in the differential abundance analysis. To estimate alpha diversity, samples were rarefied without replacement to 1,000 reads, and then observed richness and Shannon diversity (52) were calculated for each sample in phyloseq (46). Observed richness represents the number of ASVs while Shannon diversity incorporates both the number of ASVs (richness) and their relative abundance of ASVs (evenness). Wilcoxon rank-sum tests (53) were run to check for significant differences in alpha diversity metrics between Archaea and Bacteria.

Before calculating co-occurrence networks, ASV counts were set to the presence (1) or absence (0), and samples that did not contain any archaeal reads were removed. Co-occurrence networks for each species were calculated using the cooccur package (v 1.3) (54). Only co-occurrences that were significant (q < 0.05) and had a weight (absolute value of effect size) greater than or equal to 0.1 were visualized and used for further analysis. Co-occurrences between Archaea and Bacteria were visualized using igraph (v 2.0.3) (55), tidygraph (v 1.3.1)(56), and ggraph (v 2.2.1) (57).

## RESULTS

### Sequencing results

In total, 110 fecal samples from four species of birds were processed with both the Universal and Archaea-Specific primer sets, resulting in a total of 220 sequencing libraries. Of these, one Saltmarsh Sparrow sample was removed due to sequencing concerns because the number of estimated archaeal ASVs in the Universal primer data was over 10 times higher than any other sample (356 archaeal ASVs, 619 total ASVs). In an abundance of caution, we removed this sample from both data sets as we suspected amplification or sequencing error. This left 109 samples with two sequencing libraries each for a total of 218 sequencing libraries in the data set.

With the Universal data set, all 109 samples were successfully amplified and had an average of 66,987 reads and 157 ASVs per sequence library assigned to Bacteria and Archaea (Table 2). When investigating only the Archaea amplified by the Universal primers, 42 samples had at least one read assigned to Archaea with an average of 114 reads and 3 ASVs per sequence library assigned to Archaea. With the Archaea-Specific primers, reads were assigned to Archaea in 75 samples, with an average of 22,140 reads and 18 ASVs (Table 2).

**TABLE 2:** Sequencing results

	Anna’s Hummingbird	Canada Goose	Ruddy Turnstone	Saltmarsh Sparrow	Full Data set
Archaea-specific samples > 0 reads	14 (58%)	39 (100%)	19 (79%)	3 (14%)	75 (69%)
Archaea-specific reads mean and range	6,216 (208–65,588)	38,029 (1,821–129,897)	2,973 (2–8,737)	11,289 (7,803–16,908)	22,140 (2–129,897)
Archaea-specific ASVs mean and range	5 (1–30)	29 (4–84)	5 (1–22)	7 (6–9)	18 (1–84)
Universal archaea samples > 0 reads	1 (4%)	24 (62%)	11 (46%)	6 (27%)	42 (39%)
Universal archaea read mean and range	12	140 (2–865)	63 (2–595)	117 (7–241)	114 (2–865)
Universal archaea ASVs mean and range	1	2 (1–5)	4 (1–24)	4 (1–7)	3 (1–24)
Universal samples > 0 reads	24 (100%)	39 (100%)	24 (100%)	22 (100%)	109 (100%)
Universal read mean and range	47,207 (11,595–279,051)	77,653 (9,501–209,724)	32,399 (3,301–88,555)	107,389 (4,253–568,777)	66,987 (3,301–568,777)
Universal ASVs mean and range	29 (10–105)	255 (68–477)	92 (27–313)	196 (6–1,365)	157 (6–1,365)

### Relationships between archaeal abundance and diversity

**Fig 1:**
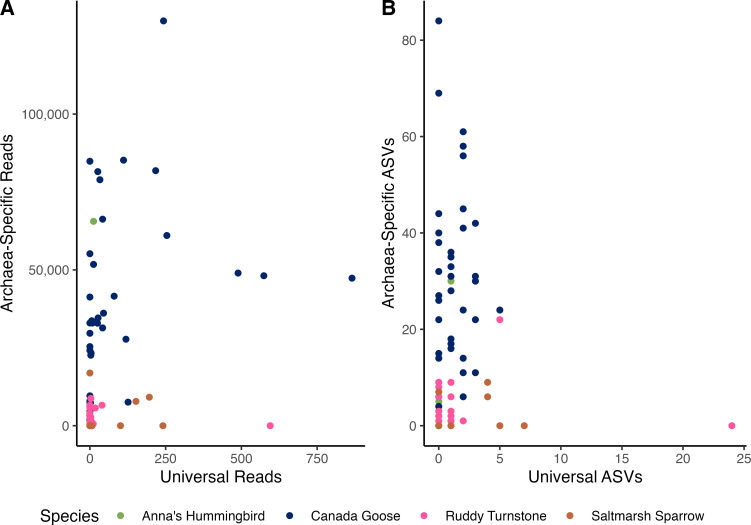
Detecting Archaea using Archaea-Specific and Universal PCR primers. (A) Number of reads assigned as Archaea using Archaea-Specific (y-axis) and Universal (x-axis) primers in 109 fecal samples from four different bird species. (B) Number of amplicon sequence variants (ASVs) assigned as Archaea using Archaea-Specific (y-axis) and Universal (x-axis) primers in fecal samples from four different bird species.

We defined the presence of Archaea as having reads assigned to Archaea and an absence of Archaea when an individual sample has no reads assigned to Archaea. Both primer sets detected the presence of Archaea in 36 samples and Archaea were absent from 28 samples using both primer sets. In 39 samples, the Archaea-Specific primers amplified archaeal reads while the Universal primers did not. The Universal primers detected the presence of Archaea when the Archaea-Specific primers did not in six samples. While the presence or absence of Archaea is sometimes corroborated by both primer sets, the number of reads found is about 200 times higher using the Archaea-Specific primers (Fig. 1A). The Archaea-Specific and Universal primers found the same number of ASVs in 30 (27.5%) samples, with 28 individuals having zero ASVs and two having 1 ASV. The Archaea-Specific primers found more ASVs in 72 (66.1%) samples, while the Universal found more ASVs in only seven (6.4%) samples (Fig. 1B).

### Community composition

#### Anna’s Hummingbird

**Fig 2:**
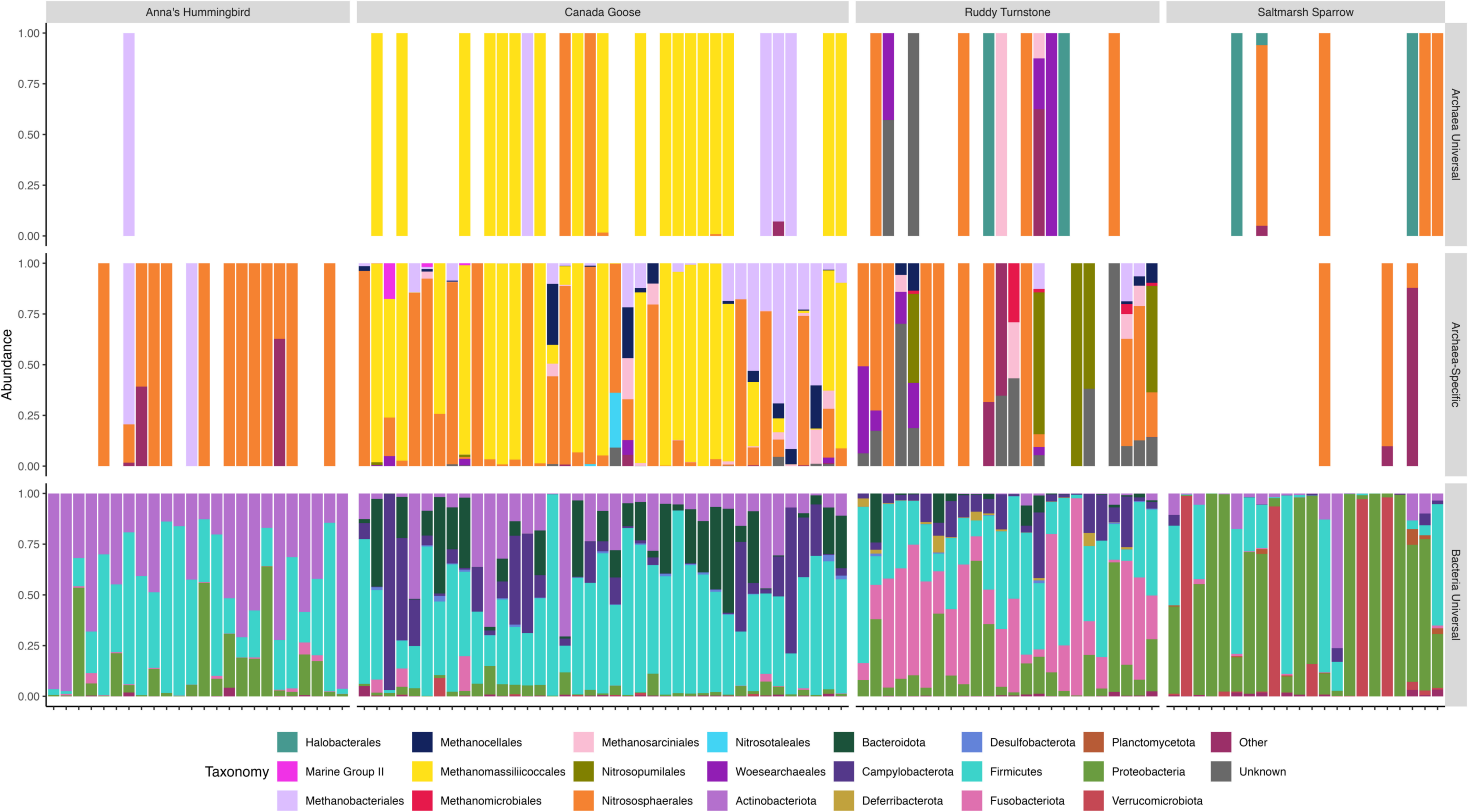
Relative abundance of archaeal orders and bacterial phyla found using Archaea-Specific and Universal PCR primers. Relative abundance of archaeal orders and bacterial phyla from Anna’s Hummingbird, Canada Goose, Ruddy Turnstone, and Saltmarsh Sparrow was found using Archaea-Specific (top) or Universal (middle and bottom) PCR primers. “Unknown” taxonomy indicates that the read was assigned to the kingdom Archaea but could not be assigned on the taxonomic level of order or that the read was assigned to the kingdom Bacteria but could not be assigned on the taxonomic level of phylum. “Other” taxonomy indicates assignment to an archaeal order or bacterial phyla that did not make up at least 1% of the sample or were not found across at least 5% of all samples.

Universal primers amplified only one archaeal genus, *Methanobrevibacter*, in the Anna’s Hummingbird samples, while the Archaea-Specific primers found six archaeal genera (Table 3). Most Anna’s Hummingbird individuals only had Archaea from the order *Nitrososphaerales;* however, other orders were also detected in a minority of samples using Archaea-Specific primers (Fig. 2). *Methanobrevibacter* was found using the Universal primers and was found in the same sample using the Archaea-Specific primers (Fig. 2). In addition, this was the most abundant genus found in the sample using the Archaea-Specific primers.

**TABLE 3:** Archaeal and bacterial taxonomic diversity

	Phyla	Classes	Orders	Families	Genera
All samples					
Archaea-specific	7	11	14	18	30
Archaea (Universal)	6	8	7	10	23
Bacteria (Universal)	30	67	177	407	1,047
Anna’s Hummingbird					
Archaea-specific	5	5	6	4	6
Archaea (Universal)	1	1	1	1	1
Bacteria (Universal)	13	18	46	77	116
Canada Goose					
Archaea-specific	5	9	13	16	21
Archaea (Universal)	3	4	3	3	3
Bacteria (Universal)	24	48	127	266	585
Ruddy Turnstone					
Archaea-specific	7	11	10	10	15
Archaea (Universal)	4	6	5	8	17
Bacteria (Universal)	24	53	134	263	408
Saltmarsh Sparrow					
Archaea-specific	3	4	4	5	5
Archaea (Universal)	2	3	2	5	6
Bacteria (Universal)	23	45	136	275	526

#### Canada Goose

In the Universal data set, few archaeal genera were recovered from Canada Goose microbiota, and most individuals’ microbiota were dominated by a single archaeal order (Fig. 2). Samples contained either order *Methanobacteriales* or order *Methanomassiliicoccales* with no overlap between the two within any individuals. Once again, the Archaea-Specific primers recovered a majority of reads from these two orders in addition to ten more archaeal orders. Almost every sample contained order *Nitrososphaerales,* and it still appeared that samples were dominated by either order *Methanobacteriales* or order *Methanomassiliicoccales*, but there was overlap in some samples. Additional methanogenic orders were also detected using Archaea-Specific primers including *Methanocellales* and *Methanosarcinales* (Fig. 2).

#### Ruddy Turnstone

Higher taxonomic diversity was found at the levels of phylum, class, and order using Archaea-Specific primers compared to Universal primers in Ruddy Turnstones (Table 3). At lower taxonomic levels (family and genus), Universal primers were found to have more diversity than Archaea-Specific primers. Universal primers typically identified a single order in samples with reads assigned to Archaea, while the Archaea-Specific primers revealed increased within-host diversity at the order level (Fig. 2). Like the previous two host species, most samples contained ammonia-oxidizing Archaea, such as order *Nitrososphaerales* or order *Nitrosopumilales*. Unlike the previous two host species, many of the Archaea-Specific reads could not be assigned to the order level and are represented by gray bars labeled Unknown in Fig. 2.

#### Saltmarsh Sparrow

Saltmarsh Sparrow samples had no appreciable difference in the amount of taxonomic diversity of Archaea found using Archaea-Specific or Universal primers (Table 3). Saltmarsh Sparrows were the only host species to have fewer samples with Archaea reads using the Archaea-Specific primers compared to the Universal primers. The orders *Halobacterales* and *Nitrososphaerales* were the most abundant orders in both the Archaea-Specific and Universal data sets (Fig. 2).

Bacterial community composition for three of the host species has been previously reported in the original publications addressing the bacterial component of their microbiomes. Anna’s Hummingbird samples had lower richness across all taxonomic levels compared to the other three host species, which displayed similar patterns to each other (Table 3). Each host species had a distinct bacterial community, but there was also overlap at the phylum level. All host species’ microbiota contained *Firmicutes* and *Proteobacteria*. The phylum *Actinobacteria* was abundant in each host species except Ruddy Turnstones, and *Campylobacterota* was common in Canada Goose and Ruddy Turnstone samples. Canada Goose samples commonly contained the phylum *Bacteroidota*, while Ruddy Turnstone samples had a high abundance of *Fusobacteriota* and several Saltmarsh Sparrow samples were dominated by the phylum *Verrucomicrobiota* (Fig. 2).

### Differential abundance of Archaea in Canada Geese

Pairwise comparisons of differential abundance between each host species only yielded significant results when involving Canada Goose samples. Most samples that produced sufficient reads using the Archaea-Specific primers (*n* = 75) were from Canada Goose (*n* = 39). In addition, there was an increased prevalence of methanogenic orders in these Canada Goose samples. This led us to test Canada Goose individuals against a grouping of all three other host species. Differential abundance analysis using ANCOM-BC2 showed two orders significantly different (df = 95; *P* < 0.05) between Canada Goose and the other three host species when compared together. These two orders are known methanogens, and one was found in higher abundance in the Canada Goose samples. *Methanomassiliicoccales* (4.90 log-fold difference, *P* = 1.2 E-6) was significantly more abundant in Canada Goose samples compared to the other three hosts, while *Methanomicrobiales* (−1.77 log-fold difference, *P* = 4.3E-2) was significantly less abundant in Canada Goose samples compared to the other three hosts.

### Alpha diversity

**Fig 3:**
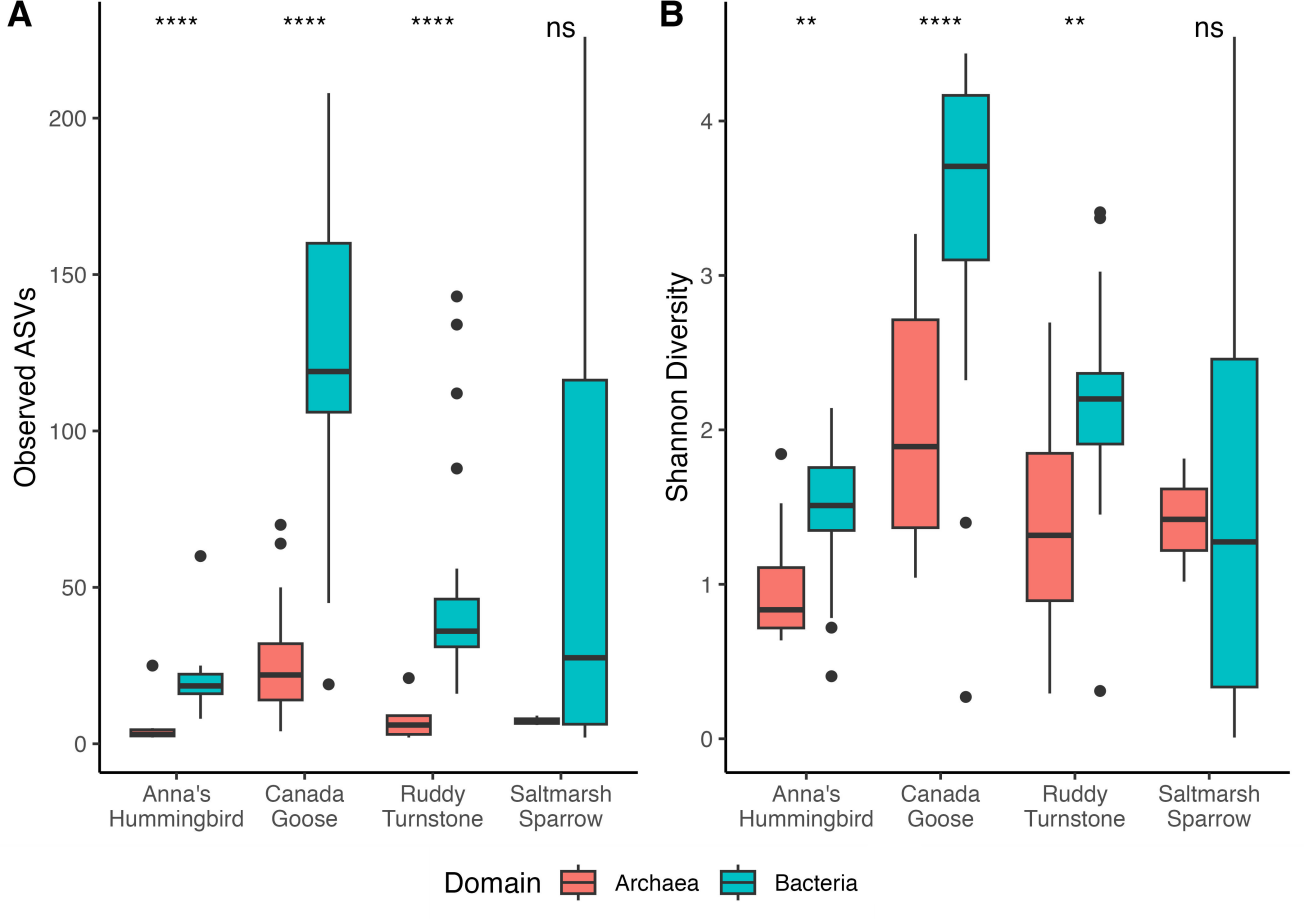
Comparing archaeal and bacterial alpha diversity. (A) Observed amplicon sequence variants (ASVs) and (B) Shannon diversity measures of Archaea and Bacteria in four bird species. All samples were rarefied without replacement to a depth of 1,000 reads. For both A and B, the middle lines on the boxes represent the median, bottom, and top lines of the box represent the first and third quartiles, respectively, and the whiskers extend to the points outside the interquartile range, up to 1.5 times the interquartile range. Dots represent points outside the range of the whiskers. Asterisks indicate significant differences in the ranked distributions of the data as determined by a Wilcoxon rank-sum test (not significant (ns): *P* > 0.05, *P ≤ 0.05, **P ≤ 0.01, ***P ≤ 0.001, ****P ≤ 0.0001).

We measured observed richness and Shannon diversity in Archaea from the Archaea-Specific data set and Bacteria from the Universal data set for four avian host species. In each host species, the number of ASVs was several times higher for Bacteria compared to Archaea (Fig. 3A); the smallest fold difference was in Anna’s Hummingbirds with only around a threefold difference, and the largest difference was in Saltmarsh Sparrows with an eightfold difference. Every host species but the Saltmarsh Sparrow had significantly (Wilcoxon rank-sum test, Anna’s Hummingbird: W = 22, *P* = 9.4E-5, Canada Goose: W = 32.5, *P* = 3.5E-13, Ruddy Turnstone: W = 2, *P* = 1.0E-6) more bacterial ASVs than archaeal ASVs. Shannon diversity was variable for each host species (Fig. 3B). Canada Geese had the largest difference in Shannon diversity comparing Bacteria (3.46) to Archaea (1.89), and Saltmarsh Sparrows had relatively equal Shannon diversity for Archaea (1.42) and Bacteria (1.47). Once again, every host species except the Saltmarsh Sparrow had significantly (Wilcoxon rank sum test, Anna’s Hummingbird: W = 55, *P* = 0.0052, Canada Goose: W = 162, *P* = 7.5E-11, Ruddy Turnstone: W = 58, *P* = 0.0013) higher Shannon diversity in the bacterial community compared to the archaeal community.

### Co-occurrence networks

Co-occurrence networks compared the presence or absence of every pair of archaeal and bacterial ASVs within all samples from a host species. Co-occurrences were then reported at the taxonomic level of phylum for Bacteria and order for Archaea to maintain consistency with other reporting in this study. Positive co-occurrences indicate that the given taxa pair is found together in individual samples more often than expected by chance, while negative co-occurrences indicate that the pair of taxa is found together in individual samples less often than expected by chance. Co-occurrences have a calculated significance and weight, and we only consider those that are significant (*P* < 0.05) and have a weight greater than 0.1. While co-occurrences themselves are not evidence of an ecological interaction, they can direct future research on ecological interactions (58). Overall, there were eight bacterial phyla and four archaeal orders that were found to have significant co-occurrences across host species, with the bacterial phylum *Firmicutes* and the archaeal order *Nitrososphaerales* being the most common taxa from their respective kingdoms.

#### Anna’s Hummingbird

**Fig 4:**
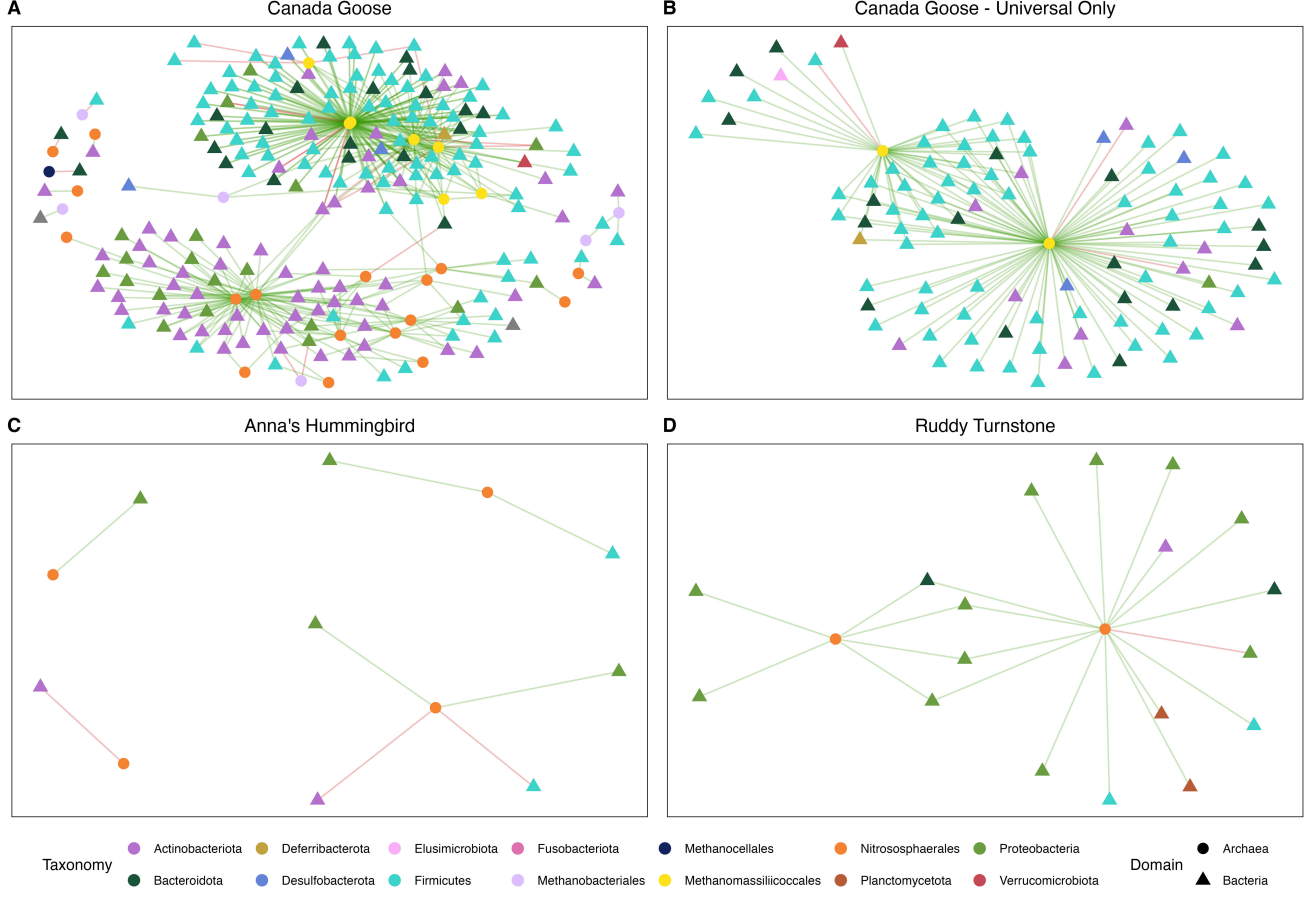
Archaea-bacteria co-occurrences. (A) Co-occurrences for Canada Goose samples using Archaea-Specific and Universal data sets. (B) Co-occurrences for Canada Goose samples using only Universal data set. (C) Co-occurrences for Anna’s Hummingbird samples using Archaea-Specific and Universal data sets. (D) Co-occurrences for Ruddy Turnstone samples using Archaea-Specific and Universal data sets. For all panels, only significant co-occurrences (*P* < 0.05) that had a weight (absolute value of effect size) >0.1 are displayed. Archaeal taxonomy is reported using orders and bacterial taxonomy is reported using phyla.

There were no Archaea-Bacteria co-occurrences in Anna’s Hummingbird when analyzing taxa found only with the Universal primer data set. When analyzing the Archaea-Specific data set and the Bacteria detected using the Universal data set, there were eight co-occurrences found. All Archaea involved were from the order *Nitrososphaerales,* and the bacterial phyla involved were either *Actinobacteria*, *Firmicutes*, or *Proteobacteria* (Fig. 4C).

#### Canada Goose

The Canada Goose samples had 134 Archaea-Bacteria co-occurrences when analyzing just the taxa found with the Universal data set (Fig. 4B). The Universal co-occurrences all involved the archaeal order *Methanomassiliicoccales,* and some of the common bacterial phyla involved were *Actinobacteria* (13/134), *Bacteroidota* (21/134), and *Firmicutes* (91/134). When analyzing the Archaea detected with the Archaea-Specific data set and the Bacteria detected using the Universal data set, there were 495 co-occurrences (Fig. 4A). The Archaea-Specific co-occurrences represented four archaeal orders and nine bacterial phyla. The most common archaeal orders were *Methanomassiliicoccales* (319/495) and *Nitrososphaerales* (164/495). The most common bacterial phyla were *Actinobacteria* (155/495), *Bacteroidota* (53/495), *Firmicutes* (234/495), and *Proteobacteria* (38/495).

#### Ruddy Turnstone

The Ruddy Turnstone samples did not have any Archaea-Bacteria co-occurrences when analyzing just the taxa found with the Universal data set. When analyzing the Archaea detected with the Archaea-Specific data set and the Bacteria detected using the Universal data set, there were 22 co-occurrences found (Fig. 4D). All Archaea involved were from the order *Nitrososphaerales*. Five bacterial phyla were involved, but most of the co-occurrences were with Bacteria from the phyla *Proteobacteria* (14/22).

#### Saltmarsh Sparrow

There were insufficient samples with sequences assigned to Archaea using either the Universal or Archaea-Specific data set to assess co-occurrence networks for Saltmarsh Sparrow samples.

## DISCUSSION

Host-associated microbiomes provide important benefits to the host organism, and even rare members of ecological communities can have important or cascading impacts (59–61). Until recently, Archaea have been largely overlooked in many studies of animal host-associated microbiomes (9–11). This study characterized archaeal diversity in the fecal microbiome of 110 individuals from four taxonomically and ecologically distinct bird species. We compared the widely used Universal 16S rRNA gene primer pair to an Archaea-Specific 16S rRNA gene primer pair and found that the Universal primers underperform in amplifying Archaea, reducing estimations of abundance and taxonomic diversity compared to the Archaea-Specific primers. We validated these results across multiple host species and found unique compositions of microbes in each host species.

### Methodological significance

We detected more Archaea when using the Archaea-Specific 16S rRNA gene primers compared to the Universal 16S rRNA gene primers. In three of the four host species, there was an increase in detection of Archaea, in both number of samples with detectable archaeal signal and archaeal richness. In the hosts with increased archaeal diversity using the Archaea-Specific primers, the Archaea identified often included those found using the Universal primers plus additional orders. For example, in Canada Goose individuals, Universal primers detected a single archaeal order in 21 out of 24 samples, and in 22 out of 24 samples the most abundant archaeal order within the Universal data set was also the most abundant order in the Archaea-Specific data set. Ruddy Turnstone did not follow this pattern, and only four out of eight samples that had archaeal reads from both primers agreed on the most abundant taxa. However, two samples had archaeal sequences from the Universal primers but not from the Archaea-Specific primers. The two Saltmarsh Sparrow samples that had archaeal sequences from both primers agreed on the most abundant taxa, but like the Ruddy Turnstones, there were four samples with archaeal sequences from the Universal primers but not from the Archaea-Specific primers. This result suggests that while the Universal primers can amplify the most abundant Archaea in a sample, they also cannot amplify the less abundant Archaea, likely due to low overall abundance in the microbiome. In addition, the fact that some samples had Archaea detected by the Universal primers but not the Archaea-Specific primers led to two hypotheses. The first is that the Archaea-Specific primers may lack sensitivity for some taxa, and the second is that Universal primers might have spurious amplicons being assigned as Archaea.

### Biological significance

#### Comparing archaeal diversity across avian hosts

Visual inspection of diversity and distribution of archaeal orders found using Archaea-Specific 16S rRNA gene PCR primers across the four host species showed widespread presence of the order *Nitrososphaerales* across the hosts and enrichment of the order *Methanomassiliicoccales* in Canada Geese compared to the other three hosts (Fig. 1). This observation was confirmed statistically using ANCOM-BC2 to show significant enrichment of *Methanomassiliicoccales* in Canada Geese compared to the other three hosts. Biological reasons for the ubiquity of *Nitrososphaerales* across the four host species and the enrichment of *Methanomassiliicoccales* in Canada Geese are explored in subsequent paragraphs. In addition, in Anna’s Hummingbirds and Saltmarsh Sparrows, it appears that Archaea are less common, and individual birds have less archaeal diversity, as compared to Canada Geese and Ruddy Turnstones. Biologically, these patterns could be affected by a variety of host factors such as size, diet, and behavior (62–66). Adult Canada Geese range in mass from 2,080 to 4,800 g (67) and Ruddy Turnstones from 99 to 160 g (68), while Saltmarsh Sparrows weigh 14 to 24 g (69) and Anna’s Hummingbirds are only 3.8 to 5.4 g (31). This order(s) of magnitude difference in mass leads to differences in excrement mass and possibly differences in microbial biomass in our samples, which may lead to discrepancies in archaeal reads (62, 63). All four species have distinct dietary preferences as explained in the introduction, which may lead to differential exposure to Archaea. In addition, Canada Geese and Ruddy Turnstones consume copious amounts of food (70–72), perhaps further increasing the likelihood of exposure to environmental Archaea. We suggest these biological explanations for our results with the large caveat of potential batch effects. The nature of this study design involved using DNA samples that were previously extracted by different researchers using similar but slightly different methods. This likely introduced batch effects that cannot be disentangled from species identity. We used both negative extraction and negative PCR controls and the R package *decontam* to best remove possible contaminants; however, other confounding factors may still exist in our data, and we caution against any strong biological conclusions about archaeal abundance and diversity when comparing these four host species (47, 73).

#### Presence of ammonia-oxidizing Archaea across avian hosts

The presence of ammonia-oxidizing Archaea across all four avian hosts was an expected result as these taxa have been previously reported to be associated with birds (68, 69). Three orders of Archaea capable of ammonia oxidization were found across all four avian host species using the Archaea-Specific primers. The most common order found was *Nitrososphaerales* in 62 out of 73 (85%) samples. Ammonia-oxidizing Archaea and Bacteria are commonly found in terrestrial and aquatic environments (74, 75) and may be associated with plant (76, 77) or animal (72) hosts. All animals produce nitrogenous waste either in the form of ammonia, urea, or uric acid (78). Birds mainly produce uric acid, which is not stored in a separate organ such as a urinary bladder and is instead exposed to a wide range of microbes in the cloaca, colon, and ceca (79). Some microbes, such as *Klebsiella aerogenes* of the bacterial phylum *Proteobacteria* (80), can utilize uric acid for their metabolism, in turn producing ammonia, carbon dioxide, and short-chain fatty acids (81). This ammonia might then be used by the ammonia-oxidizing Archaea, including those in order *Nitrososphaerales*, suggesting relevance in the four host taxa of this study and broadly across uric acid-producing birds.

#### Differential abundance of methanogenic Archaea in Canada Goose

We hypothesized that based on the natural history and lifestyles of the host species in this study, Canada Goose would have relatively higher abundances of methanogenic Archaea when compared to the other three species due to their primarily folivorous diet. When observing the relative abundance of archaeal orders found using the Archaea-Specific primers, there were more methanogenic orders in Canada Geese compared to the other three host species. While many life-history attributes differ between all four host bird species in this study, diet is of particular interest for this result. Canada Geese are herbivores and eat mainly plant matter (82, 83). Herbivory is relatively rare in birds, and many herbivorous birds are large and flightless (4, 78). Methanogenic Archaea can form syntrophic relationships with fermentative Bacteria that break down cellulose (4, 84). Examples of methanogenic Archaea in the microbiomes of herbivorous animals such as termites, ruminants, and the Hoatzin are common (18, 29, 85).

Hoatzins are folivorous birds that use foregut fermentation in their crop to acquire nutrients from their folivorous diet. By contrast, Canada Geese are avian folivores that do not use foregut fermentation for digestion. One hypothesis posits that Canada Geese and other avian folivores only extract readily available nutrients from plants as their fast mean digesta retention time, around 2 to 8 hours in closely related Barnacle Geese (*Branta leucopsis*) with seasonal variance (86), does not appear to allow for fermentation to take place (87, 88). However, previous results showed an enrichment of genes and transcripts associated with bacterial carbohydrate digestion in the microbiome of Canada Geese (83). In conjunction with our results showing enrichment of methanogenic Archaea, this suggests that Canada Geese may be able to metabolize plant molecules such as cellulose with the aid of their microbiota. In addition, the enrichment of *Methanomicrobiales* in the other three host species suggests that this methanogenic order is not likely associated with folivory in birds.

#### Relationships between bacterial and archaeal alpha diversity

We aimed to investigate if there is any relationship between bacterial and archaeal diversity within samples. When comparing only richness using the observed number of ASVs (Fig. 3A), every host species except Saltmarsh Sparrow had a significantly greater diversity of Bacteria compared to Archaea. When also accounting for relative abundance using the Shannon index (Fig. 3B), the diversity of Bacteria was again significantly greater than Archaea in every host except Saltmarsh Sparrow. The exclusion of the Saltmarsh Sparrow from having significantly more bacterial diversity than archaeal diversity is likely due to the low sample size from the Archaea samples (*n* = 3). This result supports the idea that Archaea only make up a smaller subset of the total diversity of the avian fecal microbiome compared to Bacteria in the four host species we studied.

#### Increased detection of Archaea-Bacteria co-occurrences using Archaea-specific primers

Organisms living in a microbiome exist in a community that can be characterized by the ecological interactions between members. These interactions can be difficult to untangle, especially in diverse communities. Co-occurrences of microbes in the microbiota within a host species identify patterns that may be indicative of an ecological interaction such as mutualism or competition (89). Several examples of ecological interactions between an archaeal species and a bacterial species within the human microbiome have been found, for example, transfer of H_2_ between the bacterial genus *Christensenella* and archaeal genus *Methanobrevibacter* (84), and metabolic synergy of the bacterium *Bacteroides thetaiotamicron* and the archaeon *Methanobrevibacter smithii* (90). While both studies used co-culturing techniques to verify metabolic interactions, the impetus to conduct the studies was based on previous abundance and co-occurrence data. We aimed to characterize the abundances and co-occurrences of archaeal and bacterial taxa in avian fecal microbiomes to stimulate future research into potential interdomain interactions.

In the three focal host species where the comparison could be made, many more co-occurrences between Archaea and Bacteria were detected when using the Archaea-Specific primers compared to the Universal primers (Fig. 4). Across host species, the archaeal order *Nitrososphaerales* was commonly involved in co-occurrences. The role of *Nitrososphaerales* in animal microbiomes is unclear. In previous studies, *Nitrososphaerales* and other ammonia-oxidizing Archaea were found across host animal taxa (11). Ammonia-oxidizing Archaea are most commonly associated with terrestrial environments and may simply be environmental Archaea that are transient in animal microbiomes, or these Archaea may have evolved to tolerate the animal gastrointestinal environment to improve dispersion (10). An alternative hypothesis is that these Archaea may be present in animals whose diet consists of food with high nitrogen content like invertebrates (4, 84). Future studies should aim to characterize the potential for *Nitrososphaerales* species to colonize animal gastrointestinal tracts, the potential ecological functions they have in the community, and the benefits or detriments to host fitness.

Methanogenic Archaea are better characterized in animal microbiomes than ammonia-oxidizing Archaea and are most often associated with metabolic synergy with fermentative Bacteria that break down dietary fibers (4, 90). In Canada Geese, many of the co-occurrences involved the methyl-reducing methanogenic order *Methanomassiliicoccales*. Methyl-reducing methanogens are more often associated with diets high in pectin, such as the fruit-rich diet of orangutans (91), than digestion of fiber by fermentative bacteria by CO_2_-reducing methanogens like those in the orders *Methanobacteriales* and *Methanomicrobiales* (86). However, this does not exclude the hypothesis that Canada Geese are digesting more plant fiber than previously thought since there are also members of the orders *Methanomicrobiales* and *Methanocellales* in the samples that have significant co-occurrences with bacterial genera capable of fiber degradation like *Bacteroides* (92) and *Actinomyces* (93).

Overall, more bacterial phyla were found than archaeal phyla, with *Actinobacteria*, *Bacteroidota*, *Firmicutes*, and *Proteobacteria* all having significant co-occurrences. While co-occurrences are not a perfect metric and do not always indicate an ecological interaction (94, 95), they can still be useful insights generated when cataloging prokaryotic diversity in a microbiome. A limitation of this analysis is the use of presence-absence instead of abundance metrics. Since we used two independent sequencing metrics and did not include a quantitative analysis, like qPCR, we were unable to perform abundance correlations. Future studies should aim to include qPCR of total 16S rRNA gene copies, bacterial 16S rRNA gene copies, and archaeal 16S rRNA gene copies to normalize sequencing data.

#### Archaea in animal microbiomes

The role of Archaea in the animal microbiome is contentious. Archaea appear consistently across host taxa but often in low abundance and taxonomic diversity. In the Great Apes, Archaea-Specific primers had an increased number of reads assigned as Archaea and an increased diversity of Archaea identified compared to Universal primers (10). Across 110 vertebrate species, relationships between archaeal diversity and host phylogeny and diet are detectable (10). For birds specifically, associations were with the genus *Methanothermobacter,* and it has been hypothesized that this may be due to the relatively high internal body temperature of many bird species compared to mammals (10). Our results do not corroborate these findings, but none of the host species used in this study overlap with those used in Youngblut et al. (10). Thomas et al. (11) used a nested PCR method that differs from other studies to investigate archaeal diversity in 250 animal species, including some birds, primarily sourced from captivity/zoological institutions. In general, their results agreed with Youngblut et al. (10) that host phylogeny and diet correlate with archaeal diversity. However, Thomas et al. (11) had different results and conclusions about Archaea in the bird microbiome. They found low archaeal abundance and diversity in their avian samples and posit that perhaps birds rely little on their microbiota. Our study only focused on four ecologically distinct species, which precludes us from being able to tease apart the factors which shape the archaeal diversity of the avian microbiome using phylogenetic comparative methods. However, we note substantial differences between host species, such as the enrichment of certain methanogens in Canada Goose.

We focused on fewer species to enable broader intraspecific sampling, which allowed us to have a deeper understanding of the archaeal component of the microbiota within individuals of each host species. Future studies should aim to have robust intraspecies sample sizes and a variety of species with both varied and shared ecology and taxonomy to better tease apart the influence of biotic and abiotic factors on the archaeal component of the avian microbiome.

### Future directions and recommendations to the scientific community

Archaea may be persistent members of host-associated microbiomes across diverse host taxa; however, their detection has been limited due to their low abundance and the inadequacy of Universal primers. Future studies that aim to comprehensively catalog the prokaryotic component of a microbiome should use both Universal and Archaea-Specific primers when possible. As additional host species have the bacterial component of their microbiome described, the other components (archaeal, fungal, viral, etc.) need to similarly be addressed to fully understand the diversity and function of the microbiome. Future studies in birds should avoid batch effects to better examine biotic and abiotic drivers of archaeal diversity in the microbiome. In addition, the use of qPCR to quantify the amounts of Archaea and Bacteria in samples will allow for analysis of the abundance of prokaryotes instead of just presence and absence.

## Data Availability

Sequence and metadata are available for each data set as follows: Archaea-Specific Anna’s Hummingbird 16S rRNA amplicons at SRA Accession PRJNA1219551, Archaea-Specific Canada Goose 16S rRNA amplicons at SRA Accession PRJNA1219117, Archaea-Specific Ruddy Turnstone 16S rRNA amplicons at SRA Accession PRJNA1224787, and Archaea-Specific Saltmarsh Sparrow 16S rRNA amplicons at SRA Accession PRJNA1224780. Universal Saltmarsh Sparrow 16S rRNA amplicons at SRA Accession PRJNA1218777, Universal Anna’s Hummingbird 16S rRNA amplicons at SRA Accession PRJNA659540, Universal Canada Goose 16S rRNA amplicons at SRA Accession PRJNA906628, and Universal Ruddy Turnstone 16S rRNA amplicons at https://doi.org/10.6084/m9.figshare.11337716.v1.
